# Stent-in-stent deployment above the papilla to treat malignant hepatic hilar biliary obstruction using novel fully covered multi-hole metal stent

**DOI:** 10.1055/a-2158-7776

**Published:** 2023-09-21

**Authors:** Hirotsugu Maruyama, Kojiro Tanoue, Tatsuya Kurokawa, Yoshinori Shimamoto, Yuki Ishikawa-Kakiya, Akira Higashimori, Yasuhiro Fujiwara

**Affiliations:** Department of Gastroenterology, Graduate School of Medicine, Osaka Metropolitan University, Osaka, Japan

Fully covered self-expandable metal stents (FCSEMSs) are usually used for malignant hilar biliary obstruction because they can be removed if required and provide longer stent patency by preventing tissue ingrowth 1 2 ; however, their use in the hepatic hilum carries the risk of blocking the side branches of the hepatic ducts and stent migration 3 4 5 . Furthermore, stent-in-stent techniques are unfeasible. A new FCSEMS with multiple holes was introduced to address these problems. We report successful stent-in-stent bilateral metal stent deployment using this novel FCSEMS (HANARO Biliary Multi-Hole NEO; M.I. Tech Co., Ltd, Pyeongtaek, South Korea) ( Fig. 1 ) for malignant hilar biliary obstruction.

**Fig. 1:**
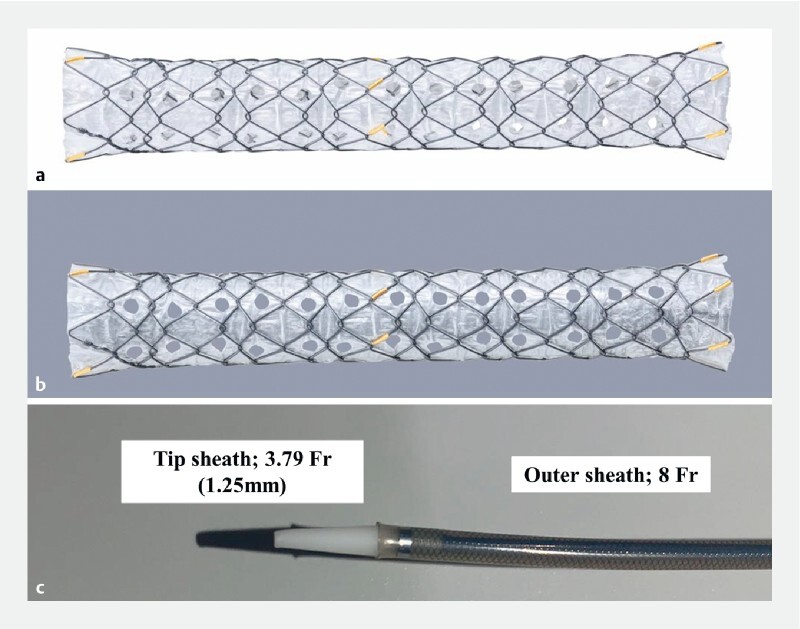
The novel covered metal stent. **a** Stent design: each side hole is 1.8 mm, and six rows are present. **b** Image highlighting side holes. **c** The diameter of the outer sheath is 8 Fr, and the tip is tapered to 3.79 Fr (1.25 mm).

A 64-year-old man with pancreatic tail cancer and liver metastases was referred because of jaundice. He was diagnosed with obstructive jaundice due to hepatic hilum metastases on computed tomography ( Fig. 2 ) followed by endoscopic retrograde cholangiopancreatography ( Video 1 ). First, we inserted a 0.025-inch guidewire into the common bile duct (CBD) and identified a Bismuth type IIIa hepatic hilar obstruction fluoroscopically. Stent-in-stent placement was performed using the novel covered metal stent. A guidewire was placed in the left and anterior bile ducts, and a stent was deployed from the left into the CBD. Another guidewire was placed from the stent lumen through a side hole and into the anterior bile duct. A second stent was deployed anteriorly into the CBD ( Fig. 3 ). The obstructive jaundice improved, and no adverse events were observed.

**Fig. 2:**
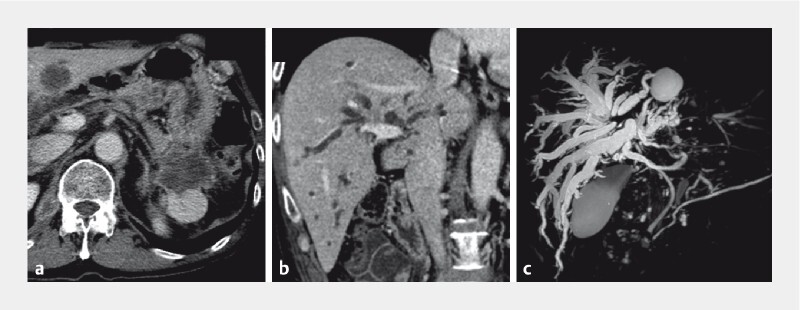
Images of the primary lesion and malignant hilar biliary obstruction. **a** A tumor with poor contrast enhancement was visible in the tail of the pancreas. The patient was diagnosed as having pancreatic cancer. **b** Metastases were present in the hepatic hilum, and the left and right intrahepatic bile ducts were dilated. **c** Magnetic resonance cholangiopancreatography showed hilar biliary obstruction and intrahepatic bile duct dilation.

**Video 1** Successful stent-in-stent deployment using newly designed covered metal stents to treat malignant hepatic hilar biliary obstruction.


**Fig. 3:**
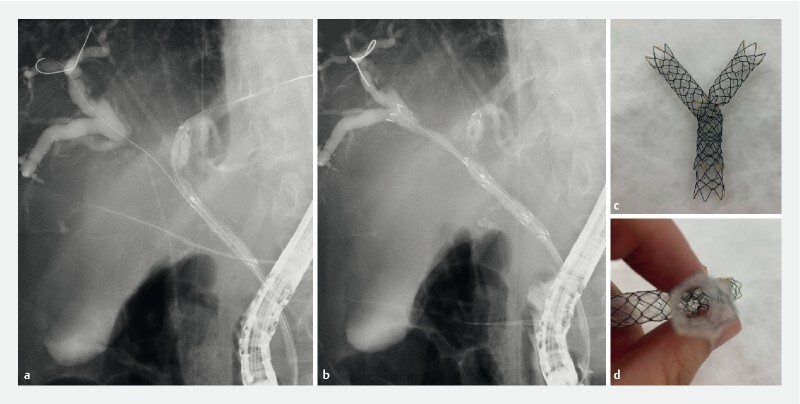
Stent placement. **a** Fluoroscopy showed Bismuth type IIIa hepatic hilar obstruction, and the novel covered metal stent was deployed from the left into the common bile duct. **b** Endoscopic bilateral stent-in-stent deployment using a novel covered metal stent. **c** In vitro observation of stent-in-stent deployment of the novel covered metal stent. **d** Stent lumen preserved.

These FCSEMSs can be inserted into contralateral bile ducts through side holes in the stent because the stent tip is tapered to 1.25 mm and it has multiple holes of 1.8 mm. The stent-in-stent technique using this stent prevents both blockage of the side branches of the hepatic ducts and tumor ingrowth. There are no previous reports of this novel technique, which may be a promising new treatment option.

Endoscopy_UCTN_Code_TTT_1AR_2AZ

## Notes

Endoscopy E-Videos

https://eref.thieme.de/e-videos
*E-Videos* is an open access online section of the journal *Endoscopy* , reporting on interesting cases and new techniques in gastroenterological endoscopy. All papers include a high-quality video and are published with a Creative Commons CC-BY license. Endoscopy E-Videos qualify for HINARI discounts and waivers and eligibility is automatically checked during the submission process. We grant 100% waivers to articles whose corresponding authors are based in Group A countries and 50% waivers to those who are based in Group B countries as classified by Research4Life (see: https://www.research4life.org/access/eligibility/ ). This section has its own submission website at https://mc.manuscriptcentral.com/e-videos
